# Targeted Lipidomic and Transcriptomic Analysis Identifies Dysregulated Renal Ceramide Metabolism in a Mouse Model of Diabetic Kidney Disease

**DOI:** 10.4172/jpb.S14-002

**Published:** 2015-05-18

**Authors:** Kelli M Sas, Viji Nair, Jaeman Byun, Pradeep Kayampilly, Hongyu Zhang, Jharna Saha, Frank C Brosius, Matthias Kretzler, Subramaniam Pennathur

**Affiliations:** 1 Division of Nephrology, Department of Internal Medicine, University of Michigan, Ann Arbor, MI, USA; 2 Department of Computational Medicine and Bioinformatics, University of Michigan, Ann Arbor, MI, USA

**Keywords:** Diabetes, Diabetic kidney disease, Diabetic nephropathy, Kidney, Ceramide, Glucosylceramide, Mass spectrometry, Lipidomics

## Abstract

Both type 1 and type 2 diabetes are associated with altered lipid metabolism, which might in part contribute to debilitating complications such as diabetic kidney disease (DKD). Ceramides are bioactive sphingolipids that have been implicated in a variety of diseases as they can regulate cellular responses to stress and invoke a myriad of downstream signaling responses. To investigate a potential role of altered ceramide metabolism in DKD, we utilized a highly sensitive and specific mass spectrometry (MS) method to quantitatively measure species in plasma and kidney cortex from the C57BLKS *db/db* mouse model of DKD and littermate controls. Long-chain ceramides (C14:0, C16:0, C18:0, C20:0) and a glucosylceramide (Glu-Cer C18:0) were increased in diabetic mouse plasma, while long-chain (C14:0, C16:0, C18:0) and very-long-chain (C24:0, C24:1) ceramides and a glucosylceramide (Glu-Cer C16:0) were decreased in diabetic mouse kidney tissue. Kidney and plasma ceramide levels correlated to functional and histopathological features of DKD. Transcriptomic analysis of mouse kidney tissue revealed expression changes indicative of decreased ceramide synthesis (Degs2, Smpd2) and increased conversion to sphingosine (Acer2) and downstream sphingosine-1-phosphate signaling. Correlation analysis identified a negative relationship between plasma and kidney tissue levels of ceramide C16:0 and ceramide C24:1. Overall, the findings suggest a previously unrecognized role for ceramide metabolism in DKD.

## Introduction

Dyslipidemia is a common feature of both prediabetes and overt diabetes mellitus. Both lack of insulin as occurring in type 1 diabetes (T1DM) and insulin resistance, which typifies type 2 diabetes (T2DM), result in altered plasma lipids in humans. High triglyceride levels that accompany either normal or impaired fasting glucose predict the development of T2DM. In addition, ~35% of T2DM adults have fasting triglyceride levels ≥ 200 mg/dL, associated with decreased high-density lipoprotein cholesterol (HDL-C) and small, dense low-density lipoprotein (LDL) particles. Patients with poorly controlled T1DM may exhibit a similar pattern of dyslipidemia [1]. Dyslipidemia has been associated with both the onset and progression of diabetic kidney disease (DKD) [2]. DKD is associated with significant morbidity and mortality as it is the most common cause of end-stage renal disease in the United States [3] and heightens cardiovascular risk [4,5]. However, the lipid components leading to DKD and its devastating outcomes are poorly understood.

Ceramides are bioactive lipids involved in a variety of cellular processes and have garnered attention for their potential involvement in a variety of diseases. Ceramides in general are known to be elevated in metabolic syndrome including obesity, diabetes, and insulin resistance [6–11]. Most ceramide studies have focused on insulin resistance and therefore the liver, muscle, and adipose tissue. Although most studies have found that plasma and tissue ceramide levels are elevated in diabetes and in metabolic syndrome, several studies have reported the opposite [12,13], conflicting the literature on ceramide levels in diabetes. Few studies have reported ceramide levels in diabetic microvascular complication-prone tissues, and most reports in DKD are extrapolated from plasma levels [12,14]. Although there are very few reports on ceramide levels in the diabetic kidney itself, results in models of acute kidney injury suggest that ceramide signaling is involved in the pathogenesis of kidney injury through increased apoptosis, TGF-β signaling and inflammation [15–20].

It is increasingly clear that not all ceramides are alike. Different ceramide species (14 to 26 carbons) have different biological functions [21]. In fact, studies suggest that overall ceramide levels may not be as indicative of function as changes in specific species [9,10]. In the case of obesity-related insulin resistance, C16:0 ceramide has been found to be critically important and promote apoptosis [9,11] and may have antagonistic physiological effects to very-long-chain ceramides such as C24:0, which is thought to be anti-apoptotic and promote proliferation. Given the conflicting literature on ceramides in kidney disease, a relative lack of information on the relationship between plasma and tissue levels, and the recent understanding that individual ceramide species are crucial to function, we investigated individual ceramide species in plasma and kidney tissue in a mouse model of DKD.

## Methods

### Reagents

All high-performance liquid chromatography grade reagents were from Sigma-Aldrich (St. Louis, MO). The ceramide standards were from Avanti Polar Lipids (Alabaster, AL).

### Animal studies

Protocols for animal use were approved by the University Committee on Use and Care of Animal of the University of Michigan and all animals were monitored by the veterinarian staff of the Unit for Laboratory Animal Medicine. Male C57BLKS *db/db* mice and littermate controls (*db/+*) were purchased from The Jackson Laboratory (Bar Harbor, ME) and used at 24 wk of age, corresponding to advanced DKD [22,23]. Mice (n = 10/group) were fasted 2 h prior to euthanasia. At time of euthanasia, plasma and kidney cortex were collected, snap frozen and stored at −80°C or preserved in paraformaldehyde.

### Tissue staining

For quantification of mesangial extracellular matrix, 3 μm sections from paraformaldehyde-fixed, paraffin-embedded kidney slices were stained using Periodic Acid-Schiff’s reagent (PAS) (n = 5/group). Mesangial area was expressed quantitatively by calculating the percentage of the total glomerular area that was PAS positive. Fifteen glomerular tufts per animal were chosen randomly for analysis. Quantification of glomerular and PAS positive areas was performed with MetaMorph Imaging Software (version 6.1) (Molecular Devices Corporation, Downingtown, PA), calibrated for the microscope and digital camera used to capture the images.

### Urine albumin and creatinine measurements

Twenty-four hour urine samples were collected in murine metabolic cages (Hatteras Instruments, Cary, NC). Quantification of albuminuria was performed by determining the urine albumin:creatinine ratio (ACR). Albuminuria was measured by plate ELISA (albuwell-M kit, Exocell/Glycadia, Philadelphia, PA) and creatinine was measured by endpoint-assay (Teco Diagnostics, Anaheim, CA). [24]

### Sample preparation for ceramide quantification by mass spectrometry

Lipids were extracted using a modified Bligh-Dyer method [25]. Lipids were extracted from 30 μl plasma or ~20 mg homogenized kidney tissue with 2:2:2 volume ratio of water:methanol:dichloromethane at room temperature after addition of the 17:0 ceramide internal standard. The organic layer was collected, dried completely under nitrogen, and resuspended in 10:90 acetonitrile:isopropanol containing 10mM ammonium acetate. Linear concentrations of a matrix-free ceramide mixture were used to monitor instrument performance and to aid in identification. Pooled samples were regularly interspersed to monitor analytical variability. The coefficient of variation (CV) for Cer C14:0, Cer C16:0, Cer C18:0, Cer C20:0, Cer C22:0, Cer C24:0, Cer C24:1, Glu-Cer C16:0, Glu-Cer C18:0 was 5.32%, 2.10%, 4.95%, 3.88%, 2.70%, 2.70%, 3.26%, 0.68%, and 1.00%, respectively.

### Targeted ceramide analysis by LC/MS

A highly-sensitive and specific liquid chromatography-electrospray ionization-tandem mass spectrometry (LC/ESI-MS/MS) method was used to monitor 12 unique ceramide species in the multiple reaction monitoring (MRM) mode [26]. Samples were subjected to MS/MS analysis using an Agilent 6410 triple quadrupole mass spectrometer coupled with an Agilent 1200 HPLC system, equipped with a multimode source (Agilent Technologies, New Castle, DE). Reverse phase LC was performed using a Waters Xbridge BEH C_18_ 2.5 μm, 2.1 × 50 mm column (Milford, MA). Mobile phase A was 5 mM ammonium acetate in water and mobile phase B was 60:40 acetonitrile:isopropanol. Positive mode LC/ESI-MS/MS was performed using the following parameters: capillary spray voltage 4000 V, drying gas flow 10 L/min, drying gas temperature 325°C and nebulizer pressure 40 psi. Flow injection analysis was used to optimize the fragmentor voltage. Optimal fragmentor voltage and cell accelerator voltage for each ceramide species in MS2 scan mode was obtained. Collision energy was optimized in product ion scan mode. Mass range between *m/z* 200 and *m/z* 800 was scanned to obtain full scan mass spectra. Individual ceramide species were detected by their characteristic LC retention time in the MRM mode. Data extraction and peak area analysis was performed using MassHunter software (version B.06.00). Concentrations were determined by comparing to the known concentration of the internal standard. Ceramide levels were normalized to plasma volume or tissue weight.

### Transcriptomics

Total RNA were extracted from mouse kidney cortex samples using the RNeasy Mini Kit (Qiagen, Hilden, Germany). Gene expression profiling were performed using Affymetrix Mouse Genome 430 2.0 arrays according to the manufacturer’s instructions. The raw image files (CEL files) were processed and normalized using Expression File Creator module implemented in Genepattern platform (http://www.broadinstitute.org/cancer/software/genepattern/). The Robust Multichip Average normalized and log2-transformed expression values were used for downstream differential analysis. Significance Analysis of Microarrays in MeV TiGR Software was used to compute the fold change differences in genes comparing the controls to the diabetic mice. Significance was assessed at an FDR of < 0.05. Downstream functional analysis of enriched pathways were generated using Ingenuity^®^ Pathway Analysis (Qiagen).

### Statistical analysis

All data were log transformed. Data analysis was performed using GraphPad Prism 6.0 (GraphPad Software, San Diego, CA). Pearson’s correlation was used to assess the relationship between plasma and kidney ceramide levels in each animal. Comparisons between groups were performed using a two-tailed student’s t-test. Significance was defined as p < 0.05.

## Results

### LC/MS/MS detection of ceramide species

**Figure 1:**
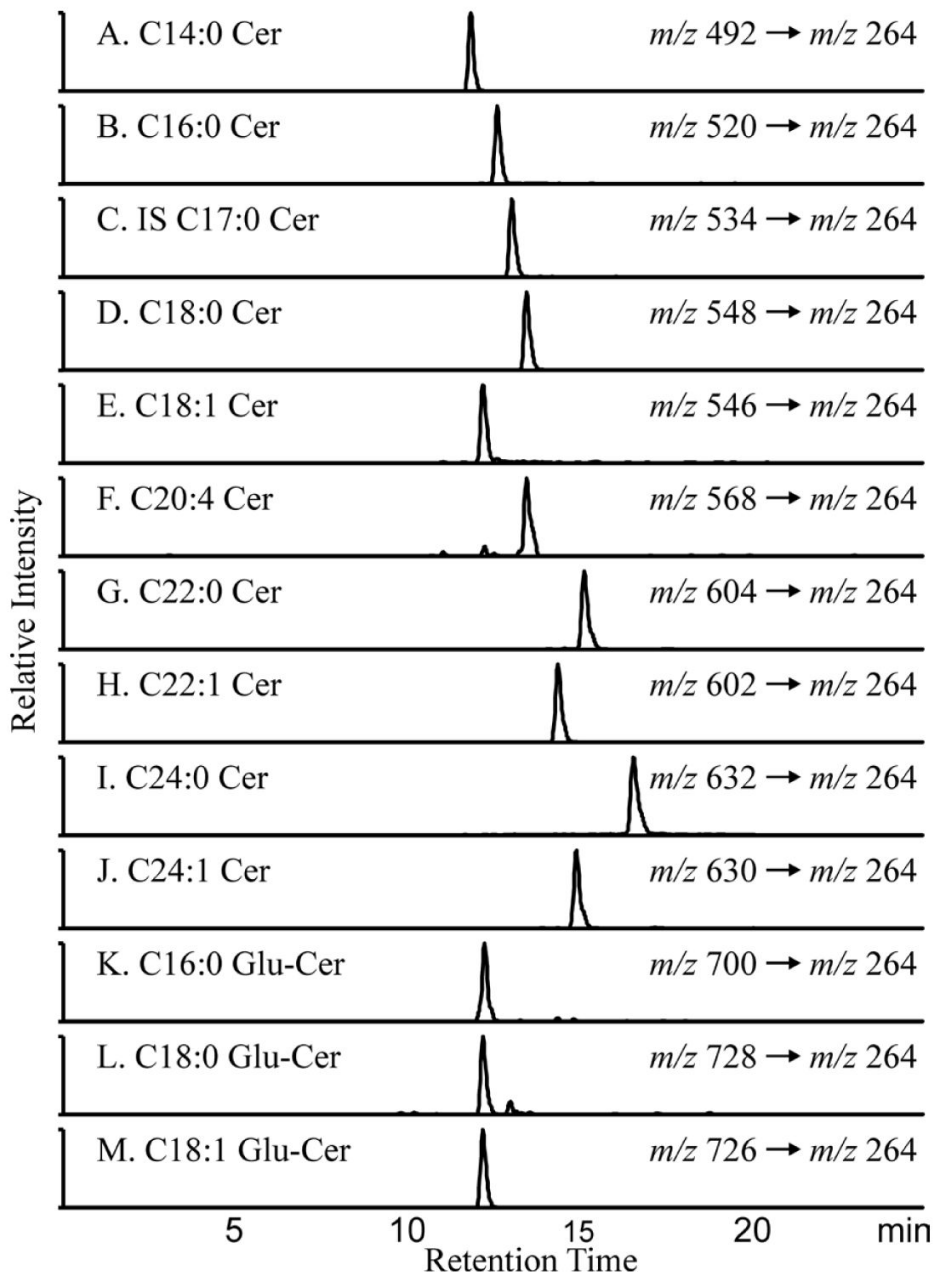
Extracted Ion Chromatograms of the measured ceramide species by LC/MS Individual ceramide species were quantified using LC/ESI-MS/MS in the MRM mode on an Agilent 6410 triple quadruple mass spectrometer. Extracted ion chromatograms and *m/z* transitions for the 12 endogenous ceramide species and the Cer C17:0 internal standard are shown. In the nomenclature (Cer C14:0), the number before the colon refers to length of the carbon chain and the number after the colon to the number of double bonds in the chain.

Plasma and kidney ceramides were quantified using LC/MS/MS in the MRM mode. Extracted ion chromatograms that were derived from the MRM transitions are shown in Figure 1. All ceramide species yield a characteristic fragment ion *m/z* 264 [(M + H) – (fatty acid chain) – H_2_O]^+^, which was used for the MRM transition. To quantify the individual subspecies, we constructed a calibration curve that used C17:0 ceramide, which was spiked into each sample as an internal standard. The ratio of ion currents for each ceramide species divided by that of the internal standard was a linear function within physiological levels of the ceramide species. The limit of detection (signal/noise > 5) was < 30 fmol for all of the species.

### Altered ceramide metabolism in plasma and kidney cortex in DKD

The concentrations of multiple ceramide species were measured in plasma and kidney cortex tissue samples from the *db/db* model (a type 2 diabetic mouse model) that develops pathologically-consistent DKD. Of the 12 ceramide species measured by the LC/MS/MS method described above, 9 were detected above the noise threshold, both in plasma and kidney cortex tissue.

**Figure 2:**
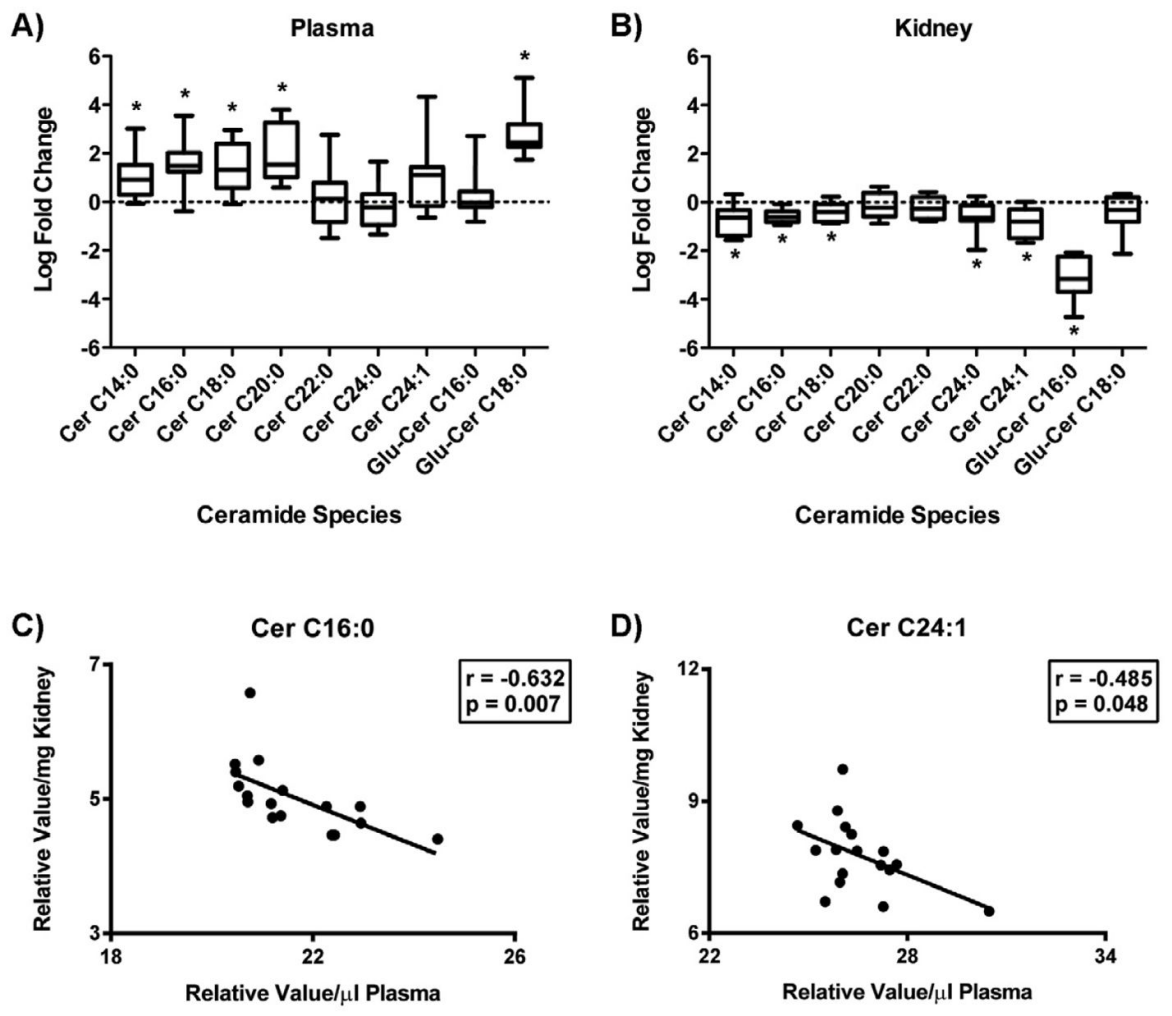
Ceramide levels in diabetic mouse plasma and kidney tissue Individual ceramide species were measured by LC/ESI-MS/MS in plasma (A) and kidney tissue (B) from 24 wk old control (*db/+*) and diabetic (*db/db*) mice by MS. Data was log2 transformed, normalized by plasma volume (μl) or tissue weight (mg), and is expressed as the relative log fold change in diabetic mice compared to control. Box-and-whiskers plots display distributions of each metabolite. The length of the box defines the interquartile range (IQR) while the whiskers denote the maximum and minimum value. The line in each box represents the median. * p < 0.05. Pearson’s correlation was used to define a relationship between plasma and kidney tissue levels per mouse. Linear regression models for ceramide species with a significant correlation (p < 0.05) are shown (C–D). In the nomenclature (Cer C16:0), the number before the colon refers to length of the carbon chain and the number after the colon to the number of double bonds in the chain.

Plasma ceramide levels were mostly elevated in diabetic mice compared to non-diabetic controls, with significance being reached for the long-chain ceramides C14:0, C16:0, C18:0, and C20:0, as well as for glucosylceramide C18:0 (Figure 2A). No significant difference was seen in the plasma abundance of very-long-chain ceramides (C22 – C24:1). Contrarily, kidney tissue ceramide species were primarily decreased in the diabetic mice compared to control mice. Significant decreases were seen in long-chain ceramides (C14:0, C16:0, C18:0), very-long-chain ceramides (C24:0, C24:1), and a glucosylceramide (Glu-Cer C16:0) (Figure 2B). Correlation analysis identified an inverse relationship between plasma and kidney tissue levels for ceramide C16:0 and ceramide C24:1 (Figures 2C and 2D), while no significant correlation was obtained for the other species measured (data not shown).

### Ceramide metabolites correlate with functional and histopathological parameters of DKD

Correlation analysis was used to examine the relationship of ceramide levels with functional and histopathological features of DKD. Albuminuria is a common indicator of glomerular disease and a feature shared between the *db/db* mouse model and human disease. Urine was collected over 24 h and urine ACR was significantly elevated in diabetic mice compared to control mice (404.6 μg/mg vs 44.4 μg/mg, p < 0.0001). Correlation analysis identified an inverse relationship between ACR and kidney tissue ceramide levels for several long-chain and very-long-chain ceramides (C14:0, 16:0, 18:0, 24:0, 24:1) and the glucosylceramides (C16:0, C18:0) (Table 1). A positive relationship was identified between plasma long-chain ceramides (C16:0, C18:0, C20:0) and a glucosylceramide (C18:0) with urine ACR (Table 1). To further examine the relationship between ceramide levels and parameters of DKD, correlations between ceramide levels and PAS staining were assessed (Table 2). PAS staining is a measure of mesangial matrix expansion, and the percentage of PAS staining in glomeruli of diabetic mice compared to control mice was significantly elevated (30.22% vs 16.97%, p < 0.0001). An inverse relationship between kidney ceramide levels and PAS staining was achieved for the glucosylceramides (C16:0, C18:0), while positive correlations were present between PAS staining and plasma levels of the long-chain ceramides (C16:0, C20:0) and a glucosylceramide (C18:0), although an inverse relationship was also identified between PAS staining and the plasma ceramide C24:0.

### Transcriptomic analysis identifies altered ceramide biosynthetic pathways in DKD

**Figure 3:**
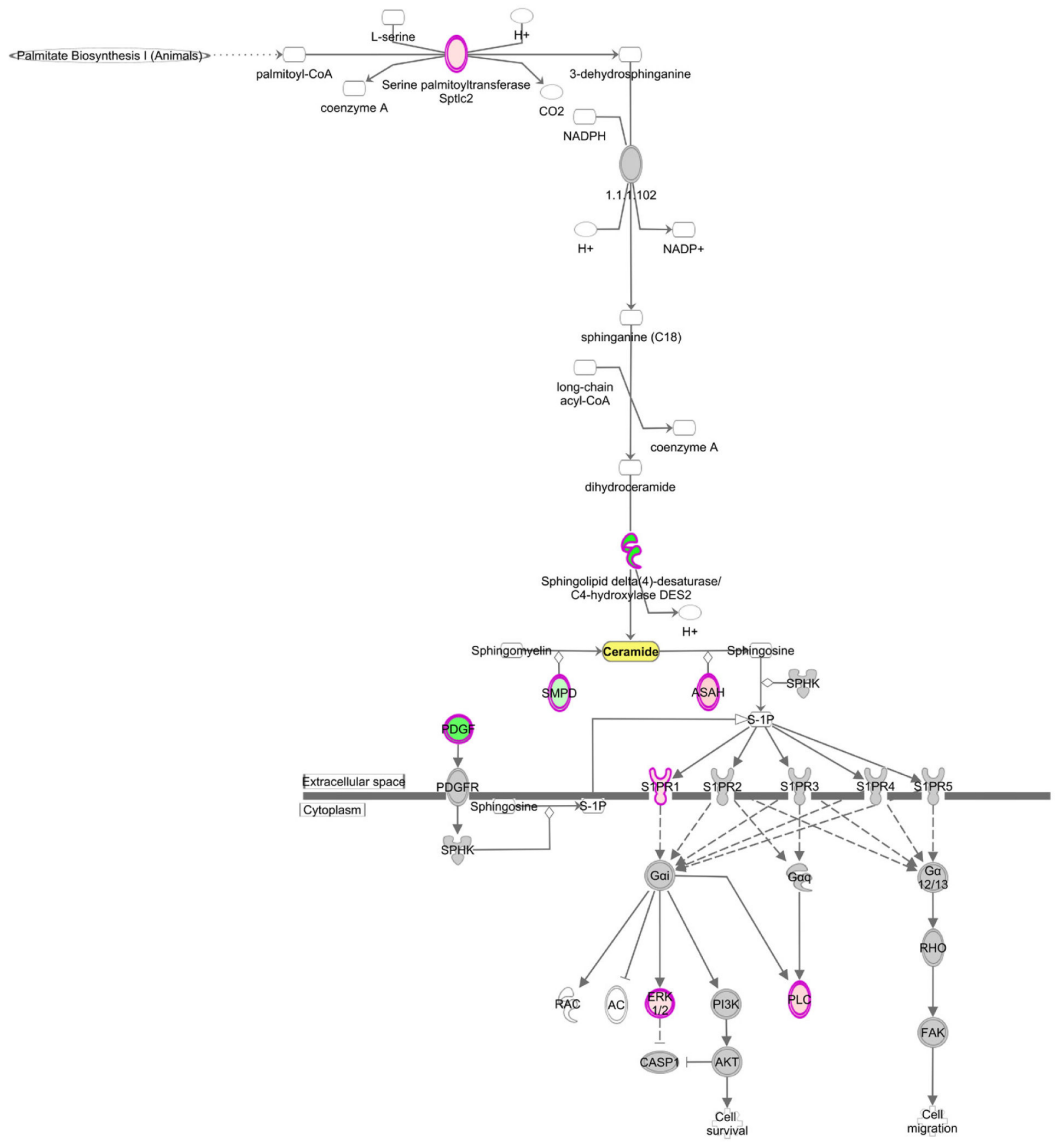
Ingenuity Pathway Analysis of ceramide synthesis and downstream signaling Transcriptomic analysis of kidney cortex from 24 wk old *db/db* diabetic mice and *db/+* control mice for genes involved in ceramide metabolism identified altered expression of genes in ceramide synthesis and downstream sphingosine-1-phosphate signaling. Ingenuity® Pathway Analysis was used to visualize changes in both the canonical ceramide biosynthesis pathway and the sphingosine-1-phosphate signaling pathway. Green = significantly downregulated, pink = significantly upregulated, gray = unchanged.

Mouse kidney transcriptomics data were analyzed for differences in gene expression of ceramide biosynthesis, degradation, and signaling. In total, 114 genes were investigated with 19 reaching significance (Table 3). Overall, the gene expression data support the conclusion that altered ceramide levels in kidney tissue were a consequence of both decreased biosynthesis as well as increased metabolism. A decrease in ceramide biosynthesis from both the *de novo* pathway and sphingomyelin metabolism was noted, as well as an increase in the conversion of ceramide to sphingosine and continued signaling through the sphingosine-1-phosphate pathway (Figure 3). Additionally, there was reduced gene expression of enzymes involved in the conversion of ceramides into glucosylceramides and galactosylceramides. While qPCR was not performed in this study, prior studies have used qPCR of differentially regulated genes and these were in agreement with the array data [27,28].

## Discussion

Diabetes is the most common cause of end-stage renal disease [3]. Although DKD is a frequent complication of both T1DM and T2DM, the physiological processes associated with DKD development are incompletely understood. Abnormal lipid metabolism has been implicated in the pathogenesis of DKD and, in the case of kidney disease, ceramides are known to be important in the response to cellular stress by promoting apoptosis and inflammation [15,29]. Previous literature regarding ceramide levels in diabetic patients and animal models has been conflicting [12,13,30], with very few reports on ceramide levels in kidney tissue. We have found that long-chain ceramides were increased in the plasma while both long-chain and very-long-chain ceramide species were decreased in kidney cortex from diabetic mice with moderate diabetic glomerulopathy.

Sphingomyelin is the second most abundant lipid associated with plasma lipoproteins [31] and can be hydrolyzed to ceramide. Patients with DKD have altered lipoprotein metabolism [32], including increased circulating levels of very-low-density lipoprotein (VLDL) and LDL [33], the primary lipoproteins with which ceramide associates [34]. Causes of hypertriglyceridemia in diabetic humans include increased hepatic VLDL production and defective removal of chylomicrons and chylomicron remnants, which often reflects poor glycemic control. The primary abnormality in DKD subjects is reduced catabolism of triglyceride rich lipoproteins, which results in elevated levels of remnant lipoproteins and prolonged postprandial hypertriglyceridemia that begins during the early stages of DKD [1]. The diminished clearance of triglyceride rich lipoproteins results from a reduction in activity of lipoprotein lipase. Many of the plasma lipid findings in humans are recapitulated in *db/db* mouse model, making it a useful model for studying diabetic dyslipidemia. Compared to controls, the *db/db* mouse has elevated plasma VLDL, LDL, triglycerides, and cholesterol and decreased lipoprotein lipase activity [35]. It is unknown if our findings of increased plasma ceramides in the diabetic mice are directly related to increased circulating levels of VLDL and LDL, but the finding has potential implications for cardiovascular health, as ceramide has been found to enhance lipoprotein aggregation and mediate the atherosclerotic effects of oxidized-LDL [36–39].

Ceramide can be generated by multiple pathways, including sphingomyelin hydrolysis, *de novo* synthesis, sphingosine recycling, or through breakdown of more complex sphingolipids [40]. In this study, kidney cortex levels of several ceramide species were decreased in diabetic mice. Although expression of serine palmitoyltransferase (Sptlc2), the rate-limiting enzyme of *de novo* synthesis, was increased, expression of a critical desaturase (Degs2) was decreased, suggesting that there could be an accumulation of dihydroceramides. Recently, an increased dihydroceramide/ceramide ratio has been linked to oxidative stress and impaired ATP synthesis [41,42], both features of diabetic kidney disease [43,44]. Although we did not measure dihydroceramides in this study, it is possible that the decrease in renal ceramide levels is due to a diminished conversion from dihydroceramides to ceramides.

Sphingolipids, including ceramide, have a rapid turnover and ceramide deacylation is the only known method of generating sphingosine, the precursor to sphingosine-1-phosphate, with about half of the generated sphingosine normally being recycled back to ceramide [45]. Transcriptomics analysis identified increased expression of the ceramidase Acer2, which would promote increased generation of sphingosine. Additionally, signaling through the downstream sphingosine-1-phosphate pathway was increased, suggesting that the decreased renal ceramide levels could be due to enhanced conversion to other sphingolipids. Indeed, previous studies have found accumulation of sphingosine-1-phosphate in the kidney cortex of diabetic rodents [46].

The BKS *db/db* mouse model used in this study is considered to be one of the best models of DKD as it has many similar features of human DKD. BKS *db/db* mice have renal hypertrophy, mesangial matrix expansion, glomerular basement thickening, tubulointerstitial changes, and albuminuria, which is accompanied by podocyte apoptosis [22,23,47,48]. We examined ceramide levels in the diabetic and control mice at 24 wk, as these pathologic features are present in the *db/db* mouse kidney at this time. Although this is considered advanced DKD in the mouse, gene expression analysis has identified this stage to be similar to early human DKD [28]. There are very few previous studies of ceramide levels and ceramide signaling in the diabetic kidney. Glucosylceramides and ceramides were found to be increased in the kidney of a streptozotocin-induced type 1 diabetes rat model and were associated with an increase in apoptosis [30,49]. One possible discrepancy between earlier findings in the type 1 streptozotocin rat model and the type 2 *db/db* mouse model is that the type 1 models were examined shortly after the onset of diabetes. Previously, ceramide levels have been shown to be transient in a model of acute kidney injury [19]. Additionally, podocyte apoptosis coincides with the onset of albuminuria, which begins at 8 wk in *db/db* mouse model. It is also possible that by examining ceramide levels in the entire renal cortex we are masking glomerular-specific changes, which is the site of fibrosis at this stage in DKD. The significant correlations between both functional and histopathological measures of DKD and ceramide levels further suggest ceramide in the glomeruli may be important in the development of DKD. The correlation between kidney cortex ceramide levels with albuminuria (a measure of glomerular disease) and increased PAS staining (mesangial expansion) suggest however that the cortical lipid content may predict pathology of glomerular and mesangial compartments as well. In support of our findings, a recent study measured total ceramide levels in kidney cortex of female *db/db* mice and reported decreased levels of total ceramides in the diabetic mice compared to controls [50]. We have expanded on this finding by reporting individual ceramide species, which reveal a global reduction in long-chain, very-long-chain, and glucosylceramide levels in the diabetic mice compared to controls.

An important finding in this study was the overall lack of an association between individual plasma ceramide levels and kidney tissue levels. It is not completely surprising given that plasma levels most likely reflect liver ceramide levels, as circulating ceramides are largely bound to lipoproteins. These findings also support the belief that *de novo* synthesis is likely to be the primary method of ceramide generation in the kidney. Palmitate is the starting point for *de novo* synthesis, so it may be that fatty acids are being shuttled towards a different pathway in the diabetic kidney. Additionally, there may be a difference in fatty acid utilization between diabetic tissues, as previous reports suggest that ceramide levels increase in diabetic liver, muscle, and adipose tissue compared to non-diabetic controls [6,10,51,52]. Regardless, these findings caution against using plasma levels as markers of tissue composition, at least in regards to ceramides in the diabetic kidney.

## Figures and Tables

**Table 1:** Correlation analysis of ceramide levels and urine ACR.

Ceramide species	Kidney Ceramides		Plasma Ceramides	
	Pearson r	p value	Pearson r	p value
Cer C14:0	−0.5968	**0.0089**	0.4498	0.0805
Cer C16:0	−0.5736	**0.0128**	0.6568	**0.0057**
Cer C18:0	−0.5964	**0.0090**	0.5802	**0.0185**
Cer C20:0	−0.3109	0.2093	0.6876	**0.0032**
Cer C22:0	−0.4414	0.0667	−0.0714	0.7928
Cer C24:0	−0.5824	**0.0112**	−0.2878	0.2797
Cer C24:1	−0.6688	**0.0024**	0.3062	0.2487
Glu-Cer C16:0	−0.9189	**<0.0001**	0.0826	0.7611
Glu-Cer C18:0	−0.4802	**0.0437**	0.8837	**<0.0001**

Pearson correlation analysis of 24 h urine albumin:creatinine ratio (ACR) with kidney ceramide levels or plasma ceramide levels from 24 wk old non-diabetic control (*db/+*) and diabetic (*db/db*) mice. Significance was defined as p < 0.05.

**Table 2:** Correlation analysis of ceramide levels and kidney PAS staining

Ceramide species	Kidney Ceramides		Plasma Ceramides	
	Pearson r	p value	Pearson r	p value
Cer C14:0	−0.4683	0.0783	0.3666	0.1790
Cer C16:0	−0.2507	0.3674	0.6493	**0.0088**
Cer C18:0	−0.2829	0.3068	0.4896	0.0640
Cer C20:0	0.0308	0.9134	0.6646	**0.0069**
Cer C22:0	−0.0926	0.7429	−0.3509	0.1997
Cer C24:0	−0.3457	0.2069	−0.5806	**0.0232**
Cer C24:1	−0.4160	0.1230	0.1945	0.4874
Glu-Cer C16:0	−0.8523	**<0.0001**	−0.2294	0.4109
Glu-Cer C18:0	−0.5305	**0.0419**	0.9215	**<0.0001**

Pearson correlation analysis of Periodic Acid-Schiff (PAS) staining in kidney sections with kidney ceramide levels or plasma ceramide levels from non-diabetic control (*db/+*) and diabetic (*db/db*) mice. Significance was defined as p < 0.05.

**Table 3:** Significantly altered genes in ceramide metabolism.

Gene Symbol	Entrez ID	Log Fold Change	q-value
Degs2	70059	−1.28	0
Ugt8a	22239	−1.07	0
Pdgfd	71785	−0.89	0.01
Mvd	192156	−0.60	0.01
Mapk14	26416	−0.42	0.02
Lass5 (Cers5)	71949	−0.38	0.01
Ugcg	22234	−0.36	0.01
Fads3	60527	−0.35	0
Smpd2	20598	−0.35	0.01
Mapk3	26417	0.27	0.02
Sptlc2	20773	0.36	0.04
Lass4 (Cers4)	67260	0.37	0.01
S1pr1	13609	0.43	0.02
Plce1	74055	0.52	0.01
Asah3l (Acer2)	230379	0.62	0
Plch1	269437	1.07	0
Plcl2	224860	1.25	0
Racgap1	26934	1.39	0.02
Smpdl3b	100340	2.83	0.02

Gene expression profiling of kidney cortex of 24 wk old *db/db* diabetic mice and *db/+* non-diabetic controls was performed using Affymetrix Mouse Genome 430 2.0 arrays. Genes involved in ceramide synthesis and metabolism were identified and significantly altered genes from kidney cortex of diabetic mice compared to non-diabetic are shown. Significance was defined as q < 0.05.

## Abbreviations

ACR: Albumin:Creatinine Ratio
Cer: Ceramide
DKD: Diabetic Kidney Disease
ESI: Electrospray Ionization
Glu-Cer: Glucosylceramide
HDL-C: High-Density Lipoprotein Cholesterol
LC: Liquid Chromatography
LDL: Low-Density Lipoprotein
MRM: Multiple Reaction Monitoring
MS: Mass Spectrometry
PAS: Periodic Acid-Schiff
T1DM: Type 1 Diabetes Mellitus
T2DM: Type 2 Diabetes Mellitus
VLDL: Very-Low-Density Lipoprotein
